# Teacher Justice and Students’ Class Identification: Belief in a Just World and Teacher–Student Relationship as Mediators

**DOI:** 10.3389/fpsyg.2018.00802

**Published:** 2018-05-23

**Authors:** Ronghuan Jiang, Ru-De Liu, Yi Ding, Rui Zhen, Yan Sun, Xinchen Fu

**Affiliations:** ^1^Beijing Key Laboratory of Applied Experimental Psychology, Institute of Developmental Psychology, Faculty of Psychology, Beijing Normal University, Beijing, China; ^2^Graduate School of Education, Fordham University, New York, NY, United States

**Keywords:** teacher justice, class identification, belief in a just world, teacher–student relationship, adolescents

## Abstract

For school-age adolescents, teacher justice plays an important role in their learning and social outcomes. The present study examined the relation between teacher justice and students’ class identification in 1735 Chinese school-age adolescents by considering belief in a just world (BJW) and teacher–student relationship as mediators. Structure equation modeling (SEM) was used to reveal the direct and indirect effects. The analyses showed that all the direct and indirect effects were significant. These findings indicated that teacher justice had a positive effect on students’ class identification. In addition, teacher justice impacted students’ class identification through students’ just-world belief and teacher–student relationships. These results suggested that for adolescents, teacher justice played an important role in shaping their just-world belief system and their interpersonal relationships with teachers, which in turn affected their sense of belonging and values in relation to their class. Thus, it is important for teachers to be aware that their injustice may negatively impact their relationships with students, students’ belief systems, and their psychological engagement at school. There is a need to develop teacher-training programs to help teachers to establish classroom reward-punishment systems with the consideration of social justice, to communicate with students through an unbiased approach, and to increase student participation in the important decision making of the whole class.

## Introduction

School plays an important role in students’ development not only because it provides opportunities for them to learn knowledge systematically, but also because it provides a collective environment that can foster their social development. In such an environment, most students will establish a unique identity related to their school life; that is, they acknowledge that they belong to a specific community in school (e.g., a particular class, sport team, junior orchestra) and are aware of the emotions and values associated with becoming a member of this community (Ashforth and Mael, 1989; Osterman, 2000; Middlebrook, 2010). Such identification is important as it has been found to affect students’ learning motivation, learning engagement, and academic performance (Faircloth and Hamm, 2005; Middlebrook, 2010; Maxwell et al., 2017) and to influence students’ development of self-identity, self-confidence, and self-esteem (Rich and Schachter, 2012; Jetten et al., 2015).

During the process of identity development, the classroom climate students experience can affect their identification with their class. According to the group engagement model, people’s social identification with a community can be influenced by their experience of being treated justly by other people (Tyler and Blader, 2000, 2003; De Backer et al., 2011). In the educational context, teachers are the stakeholders in charge of reward or punishment allocation (Resh and Sabbagh, 2014), and students’ experience of justice largely depends on teachers’ decisions and judgment. Thus, teacher justice in the classroom might be related to students’ class identification. However, only a few studies have examined the relation between experienced justice and identification in a school context (Berti et al., 2010); how these two variables are correlated and the potential intermediate paths between them remain unclear. According to previous studies, teacher justice was found to affect students’ belief in a just world (BJW) (Dalbert, 2001; Dalbert and Stoeber, 2006; Gorard, 2012) and their interactions with people around them (Paulsel and Chory-Assad, 2005; Donat et al., 2012), which might further impact students’ class identification (Bègue, 2002; Mitchell and Forsyth, 2004; Schindler and Reinhard, 2015). Therefore, the present study aims to investigate the relation between teacher justice and students’ class identification, and we consider BJW and teacher–student relationship as mediators.

### Teacher Justice and Chinese Students’ Identification With Class

Being treated fairly by teachers is important for positive student experiences in a school environment (Peter and Dalbert, 2010); such experiences also have an impact on students’ cognitive and social development (Molinari et al., 2013). Many studies have indicated that teacher justice is a key factor that can explain variance in students’ academic performance (Peter et al., 2012; Molinari et al., 2013), well-being (Kamble and Dalbert, 2012), school engagement (Berti et al., 2010; Donat et al., 2017), and school distress (Peter et al., 2013). Researchers also found that experiencing justice was correlated with many other outcomes including school belongingness and social trust (Gorard, 2011; Resh and Sabbagh, 2014). For example, Resh and Sabbagh (2014) investigated 5000 Israel middle school students and found that students’ experiences of both distributive justice and procedural justice at school positively correlated with their trust in people and formal institutions; also, such justice experiences explained students’ belongingness to their school. These results were in line with the group engagement model that emphasized links between experienced justice and attitudes toward authorities, group cooperation, and social identification (Tyler and Blader, 2003).

According to the group engagement model, the authorities (i.e., the supervisor or leader) in the group are generally viewed as representative of the whole group. In this case, the behaviors of the person in the position of authority may communicate important information, since their actions can be perceived as important indicators of the group’s opinions, attitudes, and values. When the group members are treated justly by their supervisors or leaders, they might consider themselves to be valued by the whole group. Such justice procedures and treatment then indicate a positive, respectful attitude of the members within the group and promote pride in membership. In turn, these feelings of obtained respect and pride further foster people’s group identification (Tyler and Blader, 2003; Lipponen et al., 2015). The group engagement model also suggested that people use their experienced justice as a cue for evaluating whether they can safely invest their identity with a particular group, because justice experiences provide important information about whether the group climate is helpful for them to develop and maintain a positive, satisfying group identity (Tyler and Blader, 2002; Blader and Tyler, 2009). Thus, the model proposed that justice has an impact on people’s social identity in a group.

Many studies have indicated that people’s experienced justice as an antecedent is positively correlated with their group identification (Tyler and Blader, 2000, 2003; Olkkonen and Lipponen, 2006; Blader and Tyler, 2009; Lipponen et al., 2015). For example, the results of an investigation from a sample of 160 employees indicated that both procedural and distributive justice in an organization were positively related to the employees’ organizational identification, and the supervisor-related justice was positively related to employees’ work-unit identification (Olkkonen and Lipponen, 2006). A later study with a larger sample (3370 employees) further confirmed this relation and indicated that supervisors’ justice as an antecedent predicted employees’ group identification and impacted employees’ behaviors during work (Blader and Tyler, 2009). These results suggested that whether people had been treated justly influenced their identification in the group as well as their behavior.

However, these researchers mainly focused on business settings or corporate environments. Few studies have considered school contexts, especially primary or secondary schools where teachers are usually considered as the authority in the classroom. This is because teachers are the main distributive agent who evaluates students’ performance and learning behavior, track their achievement and learning levels, and grant grades and certificates (Resh and Sabbagh, 2014). Teachers also manage the class by establishing rules and procedures, organizing the classroom settings, maintaining appropriate student behavior, and managing problem behaviors (Emmer et al., 2003). These behaviors are similar to the behaviors demonstrated by supervisors or leaders in the industry, and they also help teachers develop authority. This is especially the case in China since Chinese students usually have a head teacher who is in charge of various political, social, administrative, and daily affairs as well as academic activities. Students in a class go to the head teacher for any problems they encounter, including those that occur outside school or in classes taught by other subject teachers. In addition, the head teacher usually teaches and mentors the same group of students over several years (Chen et al., 1995; Chang et al., 2004). In such a context, teacher justice is critical for students to identify with their class, because it determines whether a student can obtain respect and esteem within the classroom, and such feelings will further affect students’ class identification (Blader and Tyler, 2009).

In fact, a previous study found that students’ sense of injustice was correlated with their psychological engagement, including their identification with their classes (Berti et al., 2010). However, this study mainly focused on classroom injustice and school engagement, and it did not investigate the potential intermediate path between students’ experienced justice and their class identification. In addition, investigating teacher justice and students’ class identification in a Chinese context will provide us with unique opportunities to understand how students’ social development could be explained by their experienced justice when they remain in a relatively stable environment over time. In China, during each stage of learning, such as elementary school, middle school, or high school, students usually stay in the same class with the same group of classmates (Li, 2015). Chinese students usually attend classes in the same home classroom during the entire academic year (i.e., subject teachers come to the home classroom to deliver instruction) and thus spend their school time with the same set of teachers and classmates in the same classroom over a long period. Many school activities (e.g., athletic competition between grades and classes) allow different classes to compete with each other, and the classes that obtain a good ranking in such activities receive a special prize in honor of the whole class, which reflects collectivism in Chinese education (Bao, 2014). In such an environment, class identification is especially important for Chinese students’ social development since it directly relates to students’ sense of belonging and pride in the class. In addition, such an environment highlights the importance of teacher justice and its influence on students’ class identification because students spend considerable time with the same set of teachers in the same classroom. Therefore, the main purpose of the present study was to investigate the relations between teacher justice and secondary school students’ class identification in a Chinese context and to further investigate the potential mediating variables.

### Belief in a Just World and Teacher–Student Relationship as Mediators

According to the just-world hypothesis, people need to believe in a just world in which people generally get what they deserve and deserve what they get (Lerner, 1965). Such belief enables people to confront the physical and social environment as stable and orderly. Although BJW was considered to be a personality disposition and unlikely to change over time (Correia et al., 2009; Faccenda and Pantaleon, 2011), many studies indicated that it was less stable in adolescence (Oppenheimer, 2005; Peter and Dalbert, 2010; Barreiro, 2013) and could be affected by educational environment (Dalbert, 2001; Dalbert and Stoeber, 2006; Thomas and Mucherah, 2016). For example, a longitudinal study found that adolescents’ personal BJW significantly increased over a period of 5 to 8 months; moreover, after controlling for age and gender, both school climate and family climate had an impact on the personal BJW (Dalbert and Stoeber, 2006). Moreover, previous studies had found that experiencing undeserved negative acts or injustice treatments (e.g., prejudice and discrimination) had a negative impact on people’s BJW (Cubela Adoric and Kvartuc, 2007; Schaafsma, 2013). These studies indicated that for school-age adolescents, their justice experiences play an important role in shaping their BJW. Since teachers are the main distributive agents and decision-makers, their just behavior is the main source of students’ justice experience. Teacher justice could affect students’ BJW, and such effect might further affect the social functions that related to BJW.

According to previous studies, one of the most important social functions of BJW is the function of trust, a function that enables people to trust others and to have confidence in having a just fate (Otto and Dalbert, 2005). Such function is critical for one’s group identification because it allows one to closely bound with group members, which might facilitate the development of membership (Tanis and Postmes, 2005). Researchers also found that strong BJW was linked to high interpersonal trust (Zuckerman and Gerbasi, 1977; Bègue, 2002; Schindler and Reinhard, 2015), and such interpersonal trust was shown to positively correlate with people’s group identification (Mitchell and Forsyth, 2004; Chen et al., 2015). Therefore, teachers’ just behavior would promote students’ BJW, and students with higher BJW would have a stronger tendency to trust their teachers and peers, while such trust in turn fosters their class identification. Besides, people with strong BJW tend to pursue long-term affiliative social goals including intimacy and altruism (Strelan, 2007; Sutton et al., 2017; Umlauft and Dalbert, 2017). As school represents an important socialization context, students’ BJW might be related to their class identification because such identification defines the social exchange relationship with others, and this process might be indispensable when they are pursuing social goals at school. A positive correlation between affiliative behavior and social identification was also found in a business setting (Liu et al., 2010). Thus, teacher justice might foster students’ BJW and further have an impact on students’ identification with their class.

In addition, teacher–student relationships could be a potential mediator in the relation between teacher justice and students’ class identification. According to previous studies, teacher justice explained students’ behaviors in the classroom such as rule-breaking and bullying (Donat et al., 2012), resistance to teachers’ requests (Paulsel and Chory-Assad, 2005), and aggression toward the teacher (Chory Assad, 2002; Chory Assad and Paulsel, 2004a,b). For example, Chory Assad and Paulsel (2004b) had found that if students perceived that the teachers used an unfair evaluation criterion when assigning grades, they tended to hold negative attitudes toward the teachers, resist teachers’ requests, and even act hostilely toward their teachers (e.g., giving negative or obscene gestures). All these behaviors are destructive to the teacher–student interaction while teacher justice appears to be an important protective factor for a positive teacher–student relationship. Researchers also found that teacher justice was correlated with students’ evaluation of interactions with their teacher; specifically, the more students experienced that they were treated fairly by their teacher, the more likely they felt their teachers were fostering a cooperative relationship instead of a hostile one (Molinari et al., 2013). These studies indicated that teacher justice played an important role in shaping a positive teacher–student relationship, and such relationship was proved to benefit the social outcomes of students, including their identity formation (Roeser et al., 2000).

Previous studies had found that students with a good teacher–student relationship tended to feel more supported and cared for (Birch and Ladd, 1997), and they tended to develop a positive attitude and sense of belonging toward their school (Zou et al., 2007; Hughes, 2011). All these positive emotions were directly linked to one’s group identification at school (Osterman, 2000; Middlebrook, 2010), indicating that a positive teacher–student relationship could foster students’ class identification. Researchers also found that for school-age adolescents, their perceptions of the quality of teacher–student relationships predicted their membership and identification with their school (Lizzio et al., 2011). These studies indicated that the effect of teacher justice on teacher–student relationship might further affect the identification of students. That is, teacher–student relationships might serve as a bridge and mediate the effect of teacher justice on students’ class identification.

### The Present Study

By taking the perspective of the group engagement model, the present study examined the associations between teacher justice and students’ class identification in the middle school context in China. The model proposed that people’s perceived justice of the authority in a group could impact their social identification with this group (Tyler and Blader, 2000), and this model was validated by many studies in the business setting (Olkkonen and Lipponen, 2006; Blader and Tyler, 2009; Lipponen et al., 2015). The present study extended the model to an educational context. We focused on school-age adolescents and eighth graders were recruited. This is because adolescence is a critical stage for one’s identity formation (Erikson, 1994; Crocetti, 2017) and early adolescents (i.e., seventh and eighth graders) tend to be more affiliated to peer groups compared to late adolescents (Gavin and Furman, 1989). In the Chinese context, eighth graders are more likely to develop the class identification compared to seventh graders because of the longer engagement in the same class (i.e., Chinese middle school students stay with the same group of classmates throughout their middle school years, including 7th, 8th, and 9th grades). Our first hypothesis is that teacher justice would have direct and positive effects on adolescents’ class identification.

In addition, although previous studies had indicated that perceived justice played an important role in students’ school identification and sense of belonging (Berti et al., 2010; De Backer et al., 2011; Resh and Sabbagh, 2014), few had considered the underlying mechanism of this relation. Thus, this study aimed to examine the mediating mechanism of the relation between teacher justice and adolescents’ class identification. Previous studies suggested that students’ BJW could be affected by their justice experience at school (Dalbert and Stoeber, 2006; Schaafsma, 2013), while students’ BJW was found to have an impact on their sense of belonging and group identification at school (Maxwell et al., 2017; Umlauft and Dalbert, 2017). Thus, we hypothesized that teacher justice would have an indirect effect on students’ class identification through BJW. Finally, researchers suggested that teacher justice could affect students’ perceived teacher–student relationship (Chory Assad and Paulsel, 2004a,b; Molinari et al., 2013), and the latter was proved to be critical in fostering students’ identification at school (Zou et al., 2007; Lizzio et al., 2011). Therefore, we further hypothesized that teacher–student relationship would mediate the effect of teacher justice on adolescents’ class identification.

## Materials and Methods

### Participants

The participants were 1735 adolescents from six middle schools in Jiangxi, China. All participants were eighth graders (mean age: 14.3 ± 0.5 years old). Among them, 900 were boys (51.8%), 827 were girls (47.7%), and 8 (0.5%) did not report their gender. The current study obtained the approval of the Research Ethics Committee of a university in Beijing and the administrative office of the participating schools. We also provided the students’ parents with written consent forms and announced that the participating students had the freedom to withdraw at any time during the course of study.

### Design and Variables

The present study used a cross-sectional design and conducted a survey-based investigation. The dependent variable is students’ class identification, and the independent variable is teacher justice. Students’ BJW and their teacher–student relationship were considered as the mediating variables. Structural Equation Model (SEM) was used to test the mediating model and the relationship among latent variables.

### Procedures

Before the investigation, students were told that all their information and their answers on the questionnaires would be used for research purposes only, and they were encouraged to answer the questions according to their true thoughts. Then, they were asked to complete a pencil-and-paper questionnaire assessing teacher justice, BJW, teacher–student relationship, and identification with class. Students took 15–20 min to complete the questionnaire and teachers were not present during the investigation. After all the students completed the questionnaire packages, we told them that they could ask their teachers for help if they needed psychological services.

### Measures

In the present study, three scales had been adapted into Chinese versions from the English version, including the teacher justice questionnaire, the teacher–student relationship questionnaire, and the class identification questionnaire. To ensure the fluency and accuracy, the items were translated by two psychology doctoral students and back-translated by two Chinese-English interpreters. Then, the translated items were reviewed by a Chinese professor of psychology and were modified to the current versions to fit the Chinese context. All items in the questionnaire package were rated on a 7-point Likert-type scale ranging from 1 (*completely disagree*) to 7 (*completely agree*). Cronbach’s alpha coefficient, McDonald’s omega coefficient, the comparative fit index (CFI), the Tucker-Lewis index (TLI), and the root mean square error of approximation (RMSEA) were reported for each instrument. We also reported the composite reliability (CR) values and the average variance extracted (AVE) of each dimension in the instruments.

#### Teacher Justice

Students responded to the teacher justice questionnaire based on their experienced justice in the classroom. The questionnaire was adopted from a cross-national study that accessed students’ experiencing justice at school (Gorard, 2012), and we adapted the questionnaire into a Chinese version. The teacher justice questionnaire included two dimensions. The first dimension measured students’ experienced teacher justice toward themselves (TJ-self) that included seven items (sample item: “I was always treated fairly by my teachers”). The second dimension measured students’ experienced teacher justice toward all students (TJ-general) that included seven items (sample item: “All students were treated the same way in class”). In this study, Cronbach’s alpha for the TJ-self, TJ-general, and the whole scale were 0.83, 0.76, and 0.89, respectively; and McDonald’s omega were 0.84, 0.76, and 0.88, respectively. The two-dimension construct validity of the scale was acceptable [χ^2^/*df* = 9.06, CFI = 0.938, TLI = 0.923, RMSEA = 0.041]. For TJ-self, CR = 0.885, AVE = 0.528, for TJ-general, CR = 0.874, AVE = 0.502.

#### Belief in a Just World

Students’ BJW was measured using an adapted BJW scale from Dalbert’s (1999) study. The adapted instrument contains six items related to BJW-self (e.g., “Overall, events in my life are just”) and six items to assess BJW-other (e.g., “I think the world is a just place for others”). The scale had been translated into a Chinese version by Jiang et al. (2013), and it was proved to be a valid and reliable tool (also see Ji et al., 2014). In the present study, Cronbach’s alpha for the BJW-self, BJW-other, and the whole scale were 0.82, 0.78, and 0.87, respectively; and McDonald’s omega were 0.84, 0.78, and 0.87, respectively. The two-dimension construct validity of the scale was acceptable [χ^2^/*df* = 9.71, CFI = 0.937, TLI = 0.920, RMSEA = 0.039]. For BJW-self, CR = 0.867, AVE = 0.523, for BJW-other, CR = 0.859, AVE = 0.509.

#### Teacher–Student Relationship

A four-item questionnaire was used to capture students’ perceived teacher–student relationship (sample item: “I get along well with my teachers”). The questionnaire was borrowed from Gorard’s (2012) study and was adapted into a Chinese version. In this study, Cronbach’s alpha for the four items was 0.68, and McDonald’s omega was 0.69. The one-dimension construct validity of the scale was acceptable [χ^2^/*df* = 8.45, CFI = 0.988, TLI = 0.963, RMSEA = 0.066], CR = 0.811, AVE = 0.519.

#### Identification With Class

The class identification questionnaire measured the extent to which students felt a sense of belonging and value in the class. It was an adaptation of the school identification questionnaire that was used in Tschannen-Moran et al.’s (2013) study. We change the word “school” to “class” and modified some sentences to fit the Chinese context. The questionnaire consisted of 10 items (sample items: “I feel proud of being part of my class”; “My classroom is one of my favorite places to be”). Cronbach’s alpha for this questionnaire was 0.83, and McDonald’s omega was 0.84. The confirmatory factor analysis on the one-dimension construct showed that the validity of the scale was acceptable [χ^2^/*df* = 12.61, CFI = 0.937, TLI = 0.914, RMSEA = 0.082]. CR = 0.895, AVE = 0.463.

### Data Analysis

We conducted descriptive analyses for all measures. We calculated Pearson correlations between each main variable. Statistical analyses of the latent variable model were conducted by using Mplus 7.0 software (Muthén and Muthén, 2012). Missing data were excluded because a sufficiently large sample was available in the present study. 1579 (91%) students provided completed data on all the variables.

In addition, we used the following criteria to evaluate the model fit: The CFI and the TLI should be equal to or greater than 0.90, and the RMSEA should be equal to or less than 0.08. In addition, a two-step procedure was conducted to examine the mediating roles of BJW and teacher–student relationship in the relation between teacher justice and class identification. First, we built a direct effect model to assess the direct effect of teacher justice on class identification. Second, we added BJW and teacher–student relationship as the mediators into the above direct path, and added predictive paths between these mediators. Finally, we conducted bias-corrected bootstrap tests with a 95% confidence interval in order to test the significance of the indirect effects.

## Results

### Descriptive Statistics and Correlations Among Main Measures

Descriptive statistics and Pearson correlations are presented in **Table 1**. The mean scores of teacher justice toward self, teacher justice toward all students, BJW-self, BJW-other, teacher–student relationship, and class identification were 5.35, 4.95, 4.94, 4.81, 4.82, and 5.65, respectively. Correlations between these variables were all positive and significant.

**Table 1:** Means, standard deviations, and correlations among main variables.

Main measures	*M ± SD*	1	2	3	4	5
(1) TJ-self	5.35 ± 1.16	1				
(2) TJ-general	4.95 ± 1.13	0.721^∗∗^	1			
(3) BJW-self	4.94 ± 1.21	0.610^∗∗^	0.655^∗∗^	1		
(4) BJW-other	4.81 ± 1.05	0.451^∗∗^	0.487^∗∗^	0.625^∗∗^	1	
(5) T–S relationship	4.82 ± 1.26	0.649^∗∗^	0.651^∗∗^	0.580^∗∗^	0.391^∗∗^	1
(6) Class identification	5.65 ± 0.96	0.640^∗∗^	0.663^∗∗^	0.663^∗∗^	0.413^∗∗^	0.640^∗∗^

*TJ, teacher justice; BJW, belief in a just world; T–S relationship, teacher–student relationship, ^∗∗^p < 0.01.*

### Examination of Mediating Effects

#### Analysis of the Direct Effect Model

Before we considered the mediating effects, we first examined the direct effect of teacher justice on class identification. The direct model was acceptable [χ^2^/*df* = 13.04, CFI = 0.932, TLI = 0.910, RMSEA = 0.057]. This result revealed that teacher justice had a positive and significant effect on class identification (β = 0.877, *p* < 0.001).

#### Analysis of the Indirect Effect Model

**FIGURE 1:**
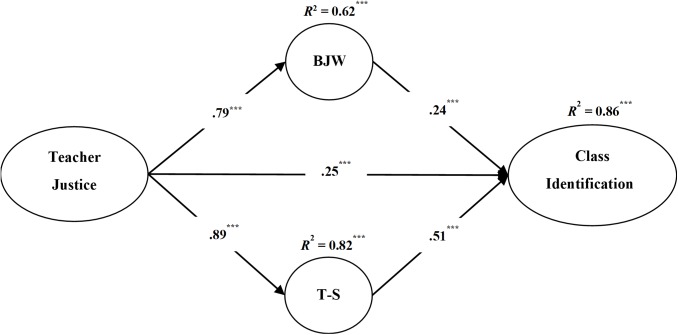
The model of mediating roles of BJW and teacher–student relationship in the relation between teacher justice and class identification. BJW, belief in a just world; T–S, teacher–student relationship. ^∗∗∗^*p* < 0.001.

Based on the direct effect model, we inserted BJW and teacher–student relationship in the relation between teacher justice and class identification (**Figure 1**). The multiple indirect effect model was also acceptable [χ^2^/*df* = 10.71, CFI = 0.908, TLI = 0.900, RMSEA = 0.075].

Finally, to evaluate the significance of the indirect effects in **Figure 1**, we conducted bias-corrected bootstrap tests with a 95% confidence interval. Five thousand bootstrap samples were created from the original data set (*N* = 1579) using random samples with replacement. The model in **Figure 1** was then conducted 5000 times with these samples to yield 5000 estimations of each indirect path coefficient. A 95% confidence interval of an indirect path coefficient that does not include 0 is suggested to be significant. As shown in **Table 2**, for example, the 95% confidence interval of the indirect path from teacher justice to class identification via BJW (0.156–0.329) did not include 0, suggesting that the indirect path was significant.

**Table 2:** Bias-corrected bootstrap test on mediating effects.

Paths	Standardized estimate (β)	95% *CI*	
		Low	High
Teacher justice—Class identification	0.246^∗∗∗^	0.034	0.563
Teacher justice—BJW—Class identification	0.189^∗∗∗^	0.156	0.329
Teacher justice—T–S—Class identification	0.459^∗∗∗^	0.370	0.845

*BJW, belief in a just world; T–S relationship, teacher–student relationship. ^∗∗∗^p < 0.001.*

## Discussion

In the present study, we aimed to investigate the relation between teacher justice and students’ class identification in a Chinese context. Moreover, we considered students’ BJW and their teacher–student relationship as the potential mediators. We found that teacher justice had a positive and direct effect on eighth graders’ identification with their class, and their BJW and teacher–student relationship partially mediated this effect.

These results are in line with the reasoning that the more students felt treated justly by their teachers, the more they felt respected, felt a sense of belonging to their class, and felt proud of being a member of the class. For students, school is the first societal environment that students confront, and it is also the place where students are exposed to authorities, obligatory rules, and collective circumstances. Therefore, students’ experience at school plays a critical role in students’ psychosocial development. In the present study, teacher justice provided important information for students to evaluate the status and legitimacy of the whole class (De Backer et al., 2011; Donat et al., 2012). That is, the more students were treated justly by their teachers, the more they viewed the class to be legitimate and in a higher status. In this case, students felt proud of being a member of the class and tended to have better identification with their class. On the other hand, teacher justice ensured that students received adequate teacher support and respect in the class, and this helped them to develop a sense of belongingness and value related to the class, which may have further fostered their class identification. This is consistent with the findings in industrial settings (Blader and Tyler, 2009; Chen et al., 2015; Lipponen et al., 2015) and it further supports the group engagement model.

Teacher justice is especially important for Chinese students to develop their class identification due to the teacher-centered approach in instructional settings. In the school setting in China, teachers were the main decision-makers in the classroom, classroom desks and tables were set up in a way that the teachers’ front desk was the center, and the arrangement represented the teachers’ authority and power (Cai and Fan, 2016). The teacher–student interaction was emphasized, and there was little peer interaction among students during the lecture time (Jiang et al., 2017). Therefore, teachers’ behaviors were the main source of students’ evaluation of the classroom climate, and whether teachers behaved justly had a great impact on students’ social outcomes. This was confirmed by the strong correlation between teacher justice and class identification in the present study. Our results also explained recent findings indicating that teacher justice could predict students’ feelings of inclusion at school and their school engagement (Donat et al., 2017; Umlauft and Dalbert, 2017). That is, the more they perceive teacher justice, the more they identify with their class, and the more they feel inclusive at school and engage in school activities.

Additionally, the present study included BJW and teacher–student relationship as potential mediators of the effect of teacher justice on students’ class identification. These results suggested that teacher justice could influence students’ identification through two paths: (a) their justice belief, and (b) their interpersonal relationship. The first path was in line with the idea that students’ experienced justice at school has an important impact on their views about what social justice is (Dar et al., 1998) and can “shape their worldviews and the ‘social map’ they construct in their mind” (Resh, 2009). Specifically, the present study assessed students’ experienced teacher justice both to themselves and to all the students; such experiences not only revealed their subjective perception of justice but also revealed the objective environment they confronted. The combination of these experiences could shape students’ just-world beliefs and further impact their class identification, which could be considered a result of the social function of BJW (Mitchell and Forsyth, 2004; Chen et al., 2015; Schindler and Reinhard, 2015). Therefore, teacher justice is important for adolescents to develop their belief system and the follow-up adaptive psychosocial functions.

The second mediating path showed that teacher justice could influence adolescents’ class identification through teacher–student relationship. This is consistent with our reasoning that teacher justice would encourage students to build a positive teacher–student relationship and such relationship would further support the formation of their “class identity”. Specifically, previous studies had shown that teacher justice was important in reducing students’ hostile behaviors such as rule-breaking and resistance to teachers’ requests (Paulsel and Chory-Assad, 2005; Donat et al., 2012); this may in turn reduce conflicts between teachers and students and help them to build a harmonious relationship. In addition, teachers’ just behaviors in the classroom ensured that all the students received assistance from the teacher, and they felt cared for and supported. Such a feeling is another key to build a positive teacher–student relationship (Hughes et al., 2001). Such a positive teacher–student relationship is also critical for students’ class identification because, on the one hand, it encourages students to develop positive attitudes toward their class and makes them feel proud of being a member of the class (Zou et al., 2007). On the other hand, it helps students to establish a sense of belonging in the class since they can obtain support and care from the teacher (Osterman, 2000). These two processes in turn will help students establish their class identification.

In general, the present study found that teacher justice can well explain variance in adolescents’ class identification through students’ BJW and the teacher–student relationship. However, these two mediators did not fully mediate the effect of teacher justice on class identification. According to the previous studies, teacher injustice was correlated with problematic behaviors among students such as bullying (Donat et al., 2012). It is possible that teacher justice affects peer relationships, and peer relationships are also an important indicator for students’ class identification (Shin et al., 2007). Thus, future studies could take this variable into account. The present study mainly focused on teacher justice toward students themselves and toward all the students in order to reflect the classroom climate, and we did not separate teacher justice into distributive, procedural, and interactional justice. However, many studies indicated that different types of justice may yield different social outcomes (Berti et al., 2010; De Backer et al., 2011; Lipponen et al., 2015). Thus, it would be interesting to investigate whether different types of teacher justice have a different relation to students’ class identification.

There are some limitations to this study. First, all our findings were based on a cross-sectional design that cannot indicate causality; we relied mainly on empirical evidence and theoretical assumptions to generate our hypotheses and to describe the predictive roles in the structural equation model. Interestingly, recent cross-sectional studies pointed out that BJW can be an antecedent variable that affects students’ perception of their justice experience (Umlauft and Dalbert, 2017; Donat et al., 2018). Thus, further longitudinal design is needed to confirm causality in the model. Second, the educational context in China is unique and different from the educational context in Western countries. Thus, the generalization of the results of this study to Western countries should be taken with caution. In the future, researchers should consider conducting similar studies in different cultural contexts to examine whether there are cultural differences. Third, the present study did not investigate further consequential variables of students’ class identification. In fact, all the variables in our study were related to important learning outcomes such as learning engagement and academic achievement (Berti et al., 2010; Middlebrook, 2010; Peter et al., 2012). It would be interesting to investigate how teacher justice affects students’ social and learning outcomes. Finally, the present study used pencil-and-paper questionnaires in the classroom setting and such data collection method cannot avoid the influence of peers during the investigation. Thus, future study could apply a computerized approach and conduct the questionnaire individually.

Nevertheless, the present study revealed that teacher justice played an important role in explaining adolescents’ class identification, with students’ just-world beliefs and teacher–student relationships as the mediators. Our results support the group engagement model and suggested that the similar pattern between experience justice and group identification could be found in school-age adolescents as well as adult employees. Furthermore, our mediating model supports this pattern and provides a detailed picture for understanding how early adolescents’ development of social identification could be influenced by their teacher. Adolescence is a critical period for one to develop their justice believes (Dalbert and Stoeber, 2006), social bonding (Forbes and Dahl, 2010), and identification (Albarello et al., 2018). Our findings indicated that besides the rapid brain and physical maturation, teacher justice as an environmental factor had strong effects on these social outcomes. These results provided important directions for establishing a positive classroom environment. For example, justice education should be involved in the teacher training and formation systems. In fact, students often consider their teachers’ grading process, honoring privileges or imposing punishment, and even the personalized student–teacher interaction itself as unjust (Peter and Dalbert, 2010; Peter et al., 2012). Thus, it is important for teachers to be aware that their injustice may negatively impact their relationships with students, students’ belief systems, students’ feeling about the whole class, and even their psychological well-being and learning engagement. There is an urgent need to develop teacher training programs to help teachers to establish classroom reward-punishment systems with the consideration of social justice, to communicate with students through an unbiased approach, and to increase student participation in the important decision making of the whole class.

## Conclusion

Our study indicated that teacher justice is an important source for adolescents to identify with their own class. Teacher justice would affect adolescents’ class identification through their just-world belief and their teacher–student relationship. Thus, schools should make particular efforts to provide a just environment and teachers should strive to behave justly toward their students to support their social development. Future studies should further investigate the causality in this mediating model by using longitudinal design and investigate the consequences of adolescents’ identification at school.

## Ethics Statement

This study was approved by the Research Ethics Committee of Beijing Normal University. All subjects gave written informed consent in accordance with the Declaration of Helsinki.

## Author Contributions

RJ collected the data, analyzed the data, and wrote the manuscript. R-DL, YD, and RZ revised the manuscript. YS and XF collected the data. All the co-authors participated in the discussion of the research results and the organization of the manuscript.

## Conflict of Interest Statement

The authors declare that the research was conducted in the absence of any commercial or financial relationships that could be construed as a potential conflict of interest.
